# Effect of Stress Management Training on Stigma and Social Phobia in HIV-Positive Women

**DOI:** 10.1177/2325958220918953

**Published:** 2020-04-10

**Authors:** Farshid Shamsaei, Neda Tahour, Efat Sadeghian

**Affiliations:** 1 Behavioral Disorders and Substance Abuse Research Center, Hamadan University of Medical Sciences, Hamadan, Iran; 2 Department of Nursing, School of Nursing and Midwifery, Hamadan University of Medical Sciences, Hamadan, Iran; 3 Chronic Diseases (Home Care) Research Centre, Hamadan University of Medical Sciences, Hamadan, Iran

**Keywords:** stress management, stigma, social phobia, women, HIV

## Abstract

The purpose of this study was to investigate the effect of stress management training on stigma and social phobia among HIV-positive women. This quasi-experimental pre- and posttest study was performed on a single group of 55 HIV-positive females Hamadan city, Iran, in 2018. The samples were taken through a convenience sampling method and the data collection tool were Berger HIV Stigma and Connor Social Phobia Scale. The mean scores of the stigma were 119.98 ± 21.15 and 94.78 ± 16.34 and social phobia were 24 ± 17.4 and 11.2 ± 9.68 before and after the intervention, respectively. The results of the paired sample *t* test indicated a significant difference in the stigma and social phobia mean scores before and after the intervention in HIV-positive women (*P* < .05). The results of the study revealed that stigma and social phobia are big challenges for HIV-positive women since these people are always judged by others and are subjected to labeling and rejection.

**What Do We Already Know about This Topic?**

Women living with human immunodeficiency virus (HIV) are highly vulnerable to poor HIV outcomes and psychological status, which are associated with nonadherence to treatment and poor engagement in HIV care.

**How Does Your Research Contribute to the Filed?**

This study was to determine the effect of stress management training on stigma and social phobia among HIV-positive women and moved further to suggest health-care measures based on the obtained results.

**What Are Your Research’s Implications toward Theory, Practice, or Policy?**

The results of this study emphasize the importance of the identification and treatment of stigma and social phobia into HIV care for women.

## Introduction

AIDS is a global health crisis, which is caused by HIV. Currently, about 36.9 million people with HIV/AIDS live in the world, 1.8 million of whom were infected with HIV in 2017. Based on the evidence, almost 75% of HIV-positive patients in the world are aware of their illness, and 21 700 000 people are on antiretroviral therapy.^1,2^

According to the national reports of Iran, up to 2017, 60 000 people of all age groups had this disease in Iran. However, only half of this population has been detected out of whom 15 000 cases were reported to be females aged above 15 years.^2^ Today, the spread of HIV and AIDS is an important health issue that has become a complex social and economic crisis.^3^ The HIV/AIDS-infected people are prone to multiple problems, such as stigma, discrimination, lack of access to services, and lack of legal rights.^4^ Stigma is experienced by both males and females; however, females are more susceptible to stigma.^5^

Stigma is a severely agonizing state that changes an ordinary person to an unimportant person with low self-esteem and self-efficacy. This state has a negative effect on the control of HIV/AIDS outbreak.^6^ As stated by Gilbert and Walker,^7^ stigma mainly occurs among the patients who receive low attention from researchers and health teams. Accordingly, stigma affects the treatment process and general health condition of patients.^7^

Psychological and emotional reactions are the other problems caused by the HIV, which can be observed in various forms of psychiatric disorders.^8^ Some of the problems reported in these patients include cognitive neurological disorders, depression, anxiety disorders (eg, social phobia and post-traumatic stress disorder), psychotic disorders, manic disorders, and personality disorders.^9^

Mental disorders can induce negative effects on the acceptance of treatment, development of high-risk behavior, and quality of life in the infected people.^8^ In a study conducted by Cook et al,^10^ the rate of anxiety disorders in HIV-positive women in the United States was reported as 61.6%.^10^ Control and management of social phobia and exposure to stigma can play an important role in the treatment and care interventions of HIV-positive patients. In this regard, stress management skills can help individuals to control themselves while facing situations, people, events, or excessive demands.^11^ Nwobi et al^12^ showed that stress management training significantly reduced the symptoms of depression, anxiety, and perceived stress. It could also improve social life satisfaction in HIV/AIDS-infected people.^12^

Stigma and social phobia regarding HIV infection among women, induced by cultural and social variables in Iran, are major health challenges that require appropriate and effective interventions. Therefore, the aim of this study was to determine the effect of stress management training on stigma and social phobia among HIV-positive women. In addition, the study moved further to suggest health-care measures based on the obtained results.

## Methods

### Design and Participants

This semi-experimental study was conducted on 55 HIV-positive women referring to Shohada Comprehensive Health Center in Hamadan city, Iran, from May to September 2017. Initially, the study population consisted of 60 HIV-positive women; however, 5 cases were excluded due to the lack of willingness to participate in the study. A comparison was made between the pre- and post-intervention results.

The inclusion criteria were (1) residency of Hamadan province; (2) 6-month history of HIV positive; (3) definitive diagnosis of the disease based on rapid test diagnostic tests, enzyme-linked immunosorbent assay, Western blot, and polymerase chain reaction; (4) absence of any psychiatric diseases; (5) lack of physical and mental disabilities; (6) lack of a stressful experience in the last month; and (7) minimum level of reading and writing literacy. On the other hand, the people who did not attend 2 more training sessions were excluded from the study.

### Data Collection

The data were collected using a demographic form, Berger HIV Stigma Scale, and Connor Social Phobia Scale. The demographic form included such variables as age, marital status, type and duration of infection, history of HIV in the family or relatives, and current occupation. The Berger HIV Stigma Scale was first designed at the Nursing Faculty, University in Chicago, Illinois, USA, during 2001 to evaluate stigma in HIV-positive patients. This scale consists of 40 items, rated based on a 4-point Likert scale ranging from strongly agree (4 points) to strongly disagree (1 point).^13^ The instrument includes 4 components, namely personalized stigma (16 items), disclosure concerns (9 items), negative self-image (8 items), and public attitudes (7 items).^14^ The total stigma score of this scale ranges from 40 to 160. Therefore, a higher score is indicative of a higher level of stigma.^15^

Toth et al confirmed the reliability of the stigma scale by test–retest correlation (0.92).^15^ In the current study, the Persian translated version of the Berger HIV Stigma Scale was used. This tool has been already used in numerous studies in Iran.^16,17^ In the present study, the reliability of the questionnaire was confirmed at a Cronbach α coefficient of 0.94.

The Connor Social Phobia Scale is a standard self-measurement scale consisting of 17 items, originally prepared by Connor et al. The questionnaire entails 3 subscales, including fear (6 items), avoidance (7 items), and physiological discomfort (4 items). This questionnaire is rated on a 5-point Likert scale ranging from very high (5 points) to very low (0 point). Therefore, the total score of an individual can be within the range of 0 to 68. According to this scale, the scores of <20, >20, and >51 are indicative of low (normal state), high, and very severe social phobia, respectively.^18^ Campbell-Sills et al confirmed the reliability of the Connor Social Phobia Scale using the test–retest method (*r* = 0.94).^19^

In the current study, the Persian translated version of the Connor Social Phobia Scale was utilized. The translated version of this instrument has been previously used in numerous studies carried out in Iran.^20,21^ In the present study, the reliability of the questionnaire was confirmed with a Cronbach α coefficient of 0.93.

### Intervention

#### Stress management training program

Stress management training interventions were conducted in 5 sessions in groups of 8 to 10 people. The training sessions were held on a weekly basis and lasted 45 to 60 minutes each. The interventions were carried out by an expert with an MSc in Psychiatric Nursing and a psychologist at Shohada Comprehensive Health Center in Hamadan, west of Iran. The stress management training program was designed and developed based on resources and texts.^2,22-25^ The summary of educational interventions is presented in the following section.

#### Content of the intervention sessions

##### Session 1

The first session was initiated by the introduction of the psychiatric nurse and participants to one another, followed by the introduction of the course plan, explanation of the purposes of the curriculum, introduction to the disease (including HIV infection, transmission, signs, and symptoms), clarification of the difference between HIV and AIDS, elucidation of HIV diagnosis, and elaboration on the consequences of HIV infection.

##### Session 2

The purposes of the second session were to present an introduction about the definition of stress, address individuals’ differences regarding stress, highlight the importance of stress management training, introduce the common effects of stress on humans, and review the impacts of stress (from physical, psychological, and behavioral aspects). Moreover, it involved a discussion about the stressors, their effects, and the probable solutions, which could be applied in the face of stress. Furthermore, relaxation training and diaphragmatic breathing were explained briefly.

##### Session 3

The session began with a review of the tasks related to the previous session. It moved further to teach stress management skills, emotional self-awareness, proper anger expression styles, anger management skills, self-expression, and time management. During this session, the participants practiced the relaxation and diaphragmatic breathing techniques.

##### Session 4

The session was initiated by a review of the tasks related to the previous session. The session continued to practice relaxation and diaphragmatic breathing, examine the existing barriers to performing tasks, study the reinforcement methods related to self-confidence, teach social skills, and practice the 7 basic steps to learn these skills (eg, active listening, frankness and honesty, self-knowledge, and awareness raising).

##### Session 5

The session was started by a review of the tasks related to the previous session. In the next step, the instructors introduced the relaxation method as a therapeutic technique, taught contraction and muscle relaxation methods, practiced the methods, resolved all ambiguous aspects regarding the application of the skills, and planned for practicing household resignation.

### Data Collection Method

The questionnaires were completed by the participants at the first and last sessions. In other words, the participants filled out the questionnaires before and after the intervention.

### Statistical Analysis

Data analysis was performed in the SPSS software (version 25) using descriptive (mean and standard deviation) and inferential statistics (paired sample *t* test). The Kolmogorov–Smirnov test was used to verify the normal distribution of data. To reject or confirm the hypotheses, the Pearson correlation coefficient was used since the normality of data were confirmed.

### Ethics Approval and Consent to Participate

This study was registered at the Iranian Registry of Clinical Trials with the code of IRCT20171206037775N1. The study was approved by the Ethics Committee of Hamadan University of Medical Sciences (IR.UMSHA.REC.1396.568). This study was conducted in accordance with the 1964 Declaration of Helsinki and its later amendments or comparable ethical standards. In this regard, the subjects were assured about the voluntariness of participation and possibility of study withdrawal at any time without facing any negative consequences. In addition, all participants provided their written informed consent.

## Results

According to the results, the mean age of the participants and mean duration of HIV infection were 38.5 ± 8.6 and 6.5 ± 3.8 years, respectively (Table 1). As indicated in Table 2, the mean scores of the stigma before and after the intervention were 119.98 ± 21.15 and 94.78 ± 16.34, respectively. The paired sample *t* test indicated a significant difference in the stigma mean scores obtained before and after the intervention in HIV-positive women (*P* < .05).

**Table 1.** Demographic Characteristics of Study Population.

Variable	N (%)
Age	
≤20	3 (5.4)
20-30	5 (9)
30-40	25 (45.4)
40-50	20 (36.3)
50≤	2 (3.64)
Marital status	
Single	9 (16.3)
Married	30 (54.5)
Divorced	3 (5.4)
Widowed	13 (23.6)
Education level	
Primary	25 (45.45)
Secondary	23 (41.8)
High	7 (12.7)
Employment status	
Employed	24 (43.6)
Unemployed	5 (9)
Housework	26 (47.2)
Illness duration (year)	
<1	1 (1.8)
1-5	14 (25.5)
5-10	34 (61.8)
10-15	3 (5.5)
>15	3 (5.5)
Route of transmission	
Blood and blood products	4 (7.2)
Needle-infected blood	4 (7.2)
Homo/bisexual	44 (80)
Mother to infant	3 (5.4)

**Table 2.** Comparison of the Mean scores of Stigma and Its Subscales Before and After the Intervention.

Subscales	Before Intervention	After Intervention		
	Mean ± SD	Mean ± SD	*t* Test	*P* Value
Personalized stigma	49.58 ± 9.73	39.16 ± 7.65	13.46	<.001
Disclosure concerns	28.01 ± 4.29	23.43 ± 4.28	10.32	<.001
Negative self-image	21.12 ± 5.15	14.69 ± 3.80	11.66	<.001
Concerns about public attitudes	21.25 ± 4.72	17.49 ± 5.33	5.72	<.001
Total stigma	119.98 ± 21.15	94.78 ± 16.34	15.17	<.001

Abbreviation: SD, standard deviation.

In addition, the mean scores of social phobia were estimated at 24 ± 17.4 and 11.2 ± 9.68 before and after the intervention, respectively, which were significantly different based on the results of the paired sample *t* test (*P* < .05; Table 3). The results also revealed a significant positive correlation between stigma and social phobia (*r* = 0.528, *P* < .01; Table 4).

**Table 3.** Comparison of the Mean Scores of Social Phobia and Its Subscale Before and After the Intervention.

Subscales	Before Intervention	After Intervention		
	Mean ± SD	Mean ± SD	*t* Test	*P* Value
Fear	8.94 ± 6.56	3.83 ± 3.47	8.54	<.001
Avoidance	10.58 ± 7.84	4.89 ± 4.22	8.73	<.001
Physiological discomfort	4.94 ± 4.08	2.47 ± 2.32	7.97	<.001
Total social phobia	24.01 ± 17.41	11.2 ± 9.68	9.73	<.001

Abbreviation: SD, standard deviation.

**Table 4.** Pearson Correlation Between Stigma and Social Phobia.

Scales	Mean ± SD	Pearson Correlation	*P* Value
Stigma	119.98 ± 21.15	0.528	<.001
Social phobia	24.01 ± 17.41		

Abbreviation: SD, standard deviation.

## Discussion

The aim of this study was to determine the effect of stress management training sessions on stigma and social phobia in HIV-positive women. The participants in this study were 55 HIV-positive women referring to the Shohada Comprehensive Health Center in Hamadan. The high mean score of stigma before the intervention indicates that stigma is one of the most important health problems in HIV-positive women.

Our findings are consistent with those of the previous studies.^15,26,27^ Amin asserted that stigma, discrimination, poor social support, violence, and mental health problems (eg, depression and neurological disorders) are big challenges for people with HIV, especially for women.^28^ Baugher et al reported that nearly 8 of every 10 people with HIV experienced stigma. In the mentioned study, the older women had higher stigma than the older men.^29^ Stigma reduces life chances and disrupts the realization of human rights.^30^ A deeper realization of stigma and mental health, as well as the accessibility of health-care systems for HIV-infected women, facilitates the implementation of stigma interventions.^31^

In most societies, attention has been paid to the issue of stigma, and it seems that stigma is a common phenomenon regardless of sociocultural differences and ethnicities.^12,32^ The results of the current study revealed that stress management skills can effectively decrease stigma in HIV-positive women. Tsai et al used a livelihood intervention to reduce stigma. Their findings were suggestive of the effectiveness of the intervention in the reduction of stigma level.^33^

Regardless of the variety of educational methods, the results showed that educational interventions are suitable methods for dealing with stigma among HIV-positive patients. In the current study, social phobia was found to be at a high level before the intervention. Social phobia was reported as a problem of the HIV-positive people in other studies.^34,35^ In a study carried out by Glémaud et al, 42.7% and 1.2% of HIV-positive women in the United States were reported to have anxiety and social phobia, respectively.^35^

Social phobia, by limiting social interactions, has a negative impact on treatment and care. Based on the evidence, people with social phobia have a strong fear of being judged by others and are embarrassed about their actions.^36^ Similar to previous studies, the results of the current study showed that stress management training sessions could reduce the mean scores of social phobia among HIV-positive women.^37,38^ The findings of the current study also revealed a relationship between stigma and social phobia. In other words, stigma and social phobia are 2 phenomena that are influenced by social and cultural factors, which are considered in many studies as the challenges for patients with HIV.^26,31,35^

Although the present study provided insightful findings, it suffered from some limitations. Firstly, there was no control group in the study due to the limitation of the research community. Secondly, training sessions were implemented for a short period of time due to the presence of personal and family problems in some patients.

### Implications

The results of this study can be used in clinics for care and treatment programs. Moreover, the health-care team can use counseling services combined with therapeutic drug interventions to provide counseling services with the aim of maintaining and improving mental health in these patients.

## Conclusion

As the results of the current study indicated, stigma and social phobia are big challenges for HIV-positive women since these people are always judged by others and are subjected to labeling and rejection. These problems originate from negative beliefs about the nature of HIV/AIDS among ordinary people in the community. Such problems can also have a negative impact on the treatment and care process. Therefore, the investigation and identification of these problems, which are often influenced by cultural and social variables, play an important role in controlling the disease. Therapeutic and care interventions, such as stress management training sessions, are among the effective methods for dealing with stigma and social phobia. These considerations can improve mental health and enhance the quality of life from various aspects.
